# Conformational flexibility of fork-remodeling helicase Rad5 shown by full-ensemble hybrid methods

**DOI:** 10.1371/journal.pone.0223875

**Published:** 2019-10-18

**Authors:** Melissa S. Gildenberg, M. Todd Washington

**Affiliations:** Department of Biochemistry, University of Iowa College of Medicine, Iowa City, Iowa, United States of America; University of Miami School of Medicine, UNITED STATES

## Abstract

Several pathways exist to bypass DNA damage during replication. One such pathway is template switching. The Rad5 protein plays two important roles in template switching: it is an E3 ubiquitin ligase that catalyzes PCNA poly-ubiquitylation and it is a helicase that converts replication forks to chicken foot structures. To understand the structure, conformational flexibility, and mechanism of Rad5, we used a full-ensemble hybrid method combining Langevin dynamics simulations and small-angle X-ray scattering. From these studies, we generated the first experimentally validated, high-resolution structural model of Rad5. We found that Rad5 is more compact and less extended than is suggested by its large amount of predicted intrinsic disorder. Thus, Rad5 likely has a novel intra-molecular interaction that limits the range of conformational space it can sample. We provide evidence for a novel interaction between the HIRAN and the helicase domains of Rad5, and we discuss the biological and mechanistic implications of this.

## 1. Introduction

**Fig 1:**
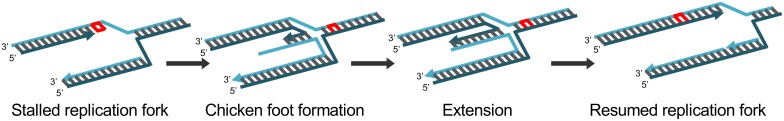
Illustration of replication fork remodeling in template switching. A stalled replication fork is shown with damage on the leading strand. Rad5 rearranges the DNA such that the stalled primer strand base pairs with the newly synthesized primer strand from the lagging strand. This results in a chicken foot intermediate. In this configuration, classical DNA polymerases can extend the stalled primer strand resulting in error-free bypass of DNA damage. Once replication has progressed beyond the site of damage, the replication fork can be reestablished and conventional DNA replication can resume.

DNA damage in the template strand blocks DNA replication by classical DNA polymerases. Cells, consequently, have evolved several pathways to bypass DNA damage during DNA replication. One pathway is translesion synthesis, which is initiated by the mono-ubiquitylation of replication accessory factor PCNA (proliferating cell nuclear antigen) [1–6]. During translesion synthesis, the stalled classical DNA polymerase is replaced by one or more non-classical DNA polymerases that catalyze DNA synthesis using the damaged strand as the template [7–16]. Typically, this is an error-prone process. Another pathway for damage bypass is template switching, which is initiated by the poly-ubiquitylation of PCNA [1–4]. During template switching, the stalled replication fork is converted to a chicken foot intermediate (Fig 1) [17,18]. Damage bypass is then accomplished by DNA synthesis using the newly synthesized sister strand as the template. Typically, this is an error-free process. In yeast, the Rad5 protein plays two critical roles in template switching. First, it binds Ubc13 (an E2 ubiquitin-conjugating enzyme) and Mms2 (a ubiquitin-conjugating enzyme variant) and acts as an E3 ubiquitin ligase to catalyze the poly-ubiquitylation of PCNA [1,2]. Second, it acts as a helicase to catalyze the conversion of the stalled replication fork to the chicken foot intermediate [17,18].

**Fig 2:**
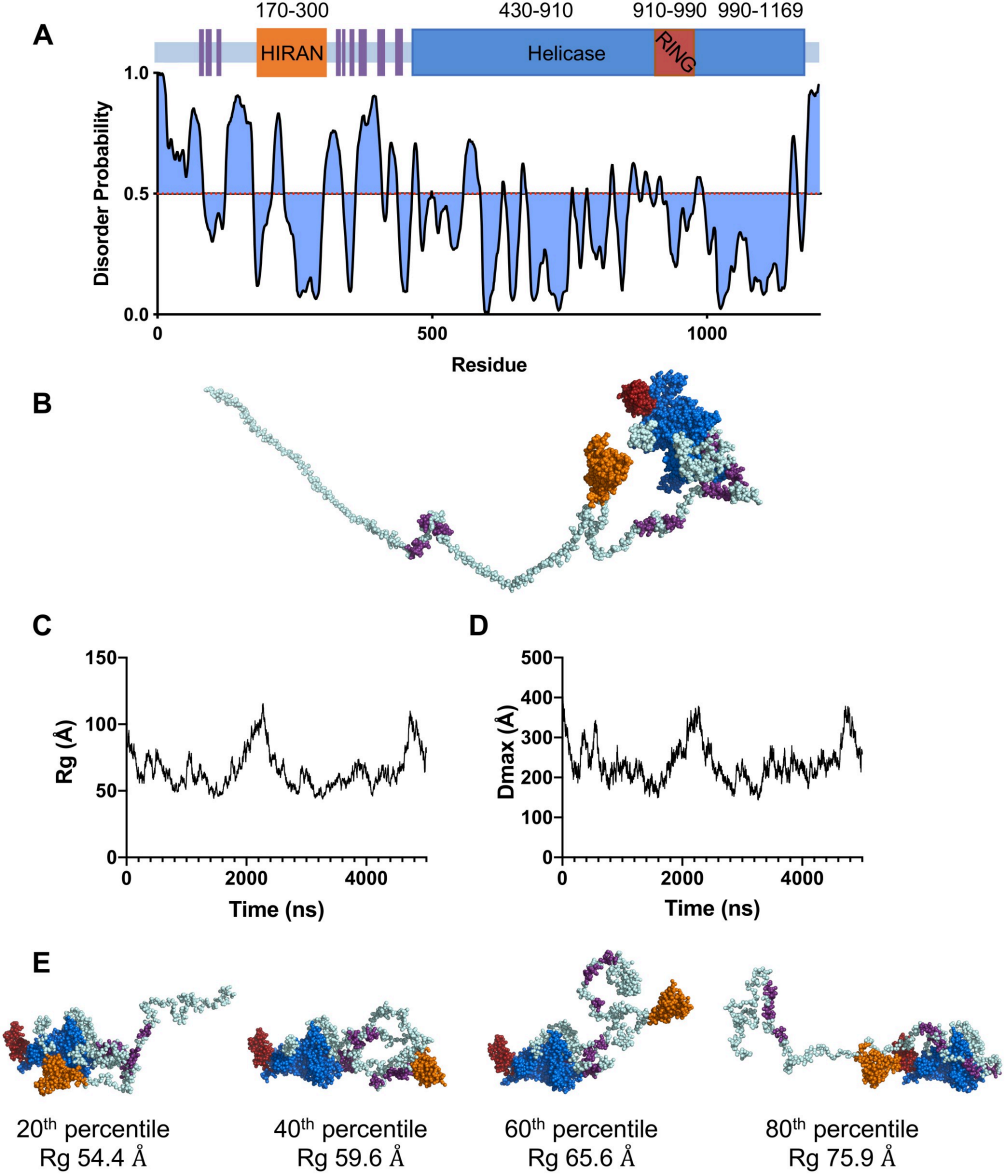
Initial LD simulations of Rad5. (**A**) Disorder probability prediction plot obtained using the PrDOS server [22,23] and an illustration of the structured and disordered regions of Rad5. Structured regions include the helicase domain (*blue*), RING finger domain (*red*), HIRAN domain (*orange*) and nine, putative α-helices (*purple*). Disordered regions are shown in *light blue*. (**B**) The starting model of Rad5 used for initial LD simulations. (**C**) R_g_ plotted over time for one 5 μs initial LD simulation. (**D**) D_max_ plotted over time for one 5 μs initial LD simulation. (**E**) Individual structures of Rad5 from the initial LD simulations representing the 20^th^, 40^th^, 60^th^, and 80^th^ percentile of R_g_ values.

Despite the critical role that Rad5 plays in DNA damage bypass, very little is known about the structure or mechanism of Rad5 or its two human homologs HLTF (helicase-like transcription factor) and SHPRH (SNF2 histone linker PHD RING helicase) [19]. Rad5 is comprised of 1169 amino acid residues that form three folded domains: a HIRAN (HIP116 Rad5p N-terminal) domain (approximately residues 170 to 300), a Swi/Snf superfamily 2 helicase domain (approximately residues 430 to 910 and 990 to 1169), and a RING (really interesting new gene) domain (approximately residues 910 to 990). The HIRAN domain, which binds free 3′ ends of DNA, and the helicase domain are both required for catalyzing fork reversal [18,20]. The RING domain and the helicase domain are required for poly-ubiquitylation of PCNA [20,21]. Only the structure of the HIRAN domain of human HLTF has been determined [18]. Protein disorder predictions indicate that the regions outside of these three domains are intrinsically disordered (Fig 2). These putative disordered regions include an N-terminal tail region (approximately residues 1 to 170) and a region between the HIRAN domain and the helicase domain (approximately residues 300 to 430). These disordered regions are likely to have high conformational flexibility.

Obtaining high-resolution structural models of proteins with high conformational flexibility is one of the most difficult challenges in contemporary structural biology. Recently, our group has successfully employed a full-ensemble hybrid method that combines Langevin dynamics (LD) simulations and small-angle X-ray scattering (SAXS) to generate experimentally validated, high-resolution structural models of several proteins with high conformational flexibility [24–26]. These included one protein with a disordered C-terminal tail region, non-classical DNA polymerase eta (pol η), as well as two proteins with short tether regions: ubiquitin-modified PCNA and SUMO (small ubiquitin-like modifier)-modified PCNA. In these cases, the LD simulations were used to generate large ensembles of several thousand structures, and these full ensembles were compared to experimental SAXS data. This was all done without resorting to curve fitting in order to avoid over fitting the experimental data.

In order to better understand the structure, conformational flexibility, and mechanism of Rad5, we have utilized this full-ensemble hybrid method. Initial LD simulations of Rad5 resulted in an ensemble in which the predicted radius of gyration (R_g_) and maximal distance (D_max_) values were significantly greater than those obtained from the experimental SAXS data. To obtain better agreement with the experimental data, we postulated a novel interaction between the HIRAN domain and the helicase domain. Including this interaction in the simulations resulted in an ensemble in which the predicted R_g_ and D_max_ values closely matched the experimental values. Thus, we have generated an experimentally validated structural model of Rad5 that has high conformational flexibility, yet retains a large, folded core comprised of the HIRAN, helicase, and RING domains.

## 2. Materials and methods

### 2.1. Protein expression and purification

Rad5 was codon-optimized for bacterial expression and cloned into pET11a with an N-terminal 6xHis tag and a C-terminal Twin-Strep tag, resulting in plasmid pKW746. Rad5 was over-expressed in BL21 Star (DE3) cells by induction at an OD_600_ of 0.6 with 1 mM IPTG for 16 hours at 16°C. Cells were lysed at 4°C using an EmulsiFlex (Avestin) in the presence of 1 mM PMSF, Complete, EDTA-free Protease Inhibitor Cocktail (Roche), and DNase. The crude extracts were clarified by ultracentrifugation. The proteins were purified using a Strep-Tactin XT resin (IBA) in 100 mM Tris pH 8.0, 150 mM KCl, 5% glycerol, and 1 mM DTT. Rad5 was eluted with 50 mM biotin and further purified using a HiLoad Superdex 200 size-exclusion column (GE Healthcare).

### 2.2. Small-angle X-ray scattering

SAXS data were collected at the BioCAT beamline 18-ID at the Advanced Photon Source. In-line size exclusion chromatography was performed using a Superdex 200 Increase 10/300 GL column, which was run at 0.7 ml/min by an AKTA Pure FPLC instrument (GE Healthcare Life Sciences). The eluate was passed through the UV monitor and through a 100 μL quartz flow cell and exposed to the X-ray beam every 2 s with 0.5 s exposures. Data were collected at room temperature using a wavelength equal to 1.033 Å, a Pilatus3 1M detector (Dectris), and a sample-to-detector distance equal to 3.5 m. Buffer subtraction was performed using BioXTAS RAW [27]. PRIMUS and AUTORG were used to calculate R_g_ values [28,29], and GNOM was used to generate D_max_ values and pair-wise distribution plots [29,30].

### 2.3. Construction of starting models for simulations

The starting model of Rad5 was built using homology models of the HIRAN domain (SWISS-MODEL, based on 4XZG.pdb) [18], RING domain (SWISS-MODEL, based on 4R8P.pdb) [31], and helicase domain (Phyre2, based on 1Z3I.pbd, 6GEJ.pbd, 3MWY.pbd, 5O9G.pbd, 6FML.pbd, and 6G7E.pbd) [32–37]. Nine putative α-helices within the intrinsically disordered regions of Rad5 were also identified and modeled using Phyre2 [38]. For our refined simulations, the ClusPro and ZDOCK docking servers were used to determine reasonable orientations of the HIRAN domain docked to the helicase domain [39,40]. Intrinsically disordered loops were built in PyMol or generated as unstructured regions in the Phyre2 models. The positions of these disordered regions were adjusted to accommodate different HIRAN domain positions using PyMol. Models were coarse-grained such that each amino acid was replaced by one to four pseudoatoms depending on the size, shape, and charge of the residue as described previously [41].

### 2.4. Langevin dynamics simulations

All simulations were carried out using the simulation code *uiowa_BD* [24]. The partial charges of the ionizable groups, the hydrodynamic radii of the pseudo-atoms, and the energy function used in the simulations were as described previously [24]. A time step of 125 fs was used, and snapshots (PDB files) were recorded after every ns of simulation time for a total of 5 μs of simulation time. This yielded an ensemble of 5,000 individual structures per simulation.

### 2.5. Comparisons of the SAXS data and the simulations

Each simulation produced an ensemble of 5,000 structures that were output as sequential PDB files. Predicted scattering curves for each of the individual structures were generated using CRYSOL [42]. The predicted scattering curve for the full ensembles were obtained by averaging the scattering curves of the individual structures constituting each of the ensembles. χ^2^ values were determined by comparing the predicted scattering curves to the experimental scattering curves as described [24]. The predicted P(r) plots for the full ensembles were obtained by summing all of the inter-atomic distances in all of the individual structures constituting each of the ensembles and generating histograms in GraphPad Prism. The cut off for determining D_max_ was set to include 99% of the inter-atomic distances.

### 2.6. Chemical crosslinking reactions

A 100-μl reaction containing 50 μM of full length Rad5 was treated with BS^2^G and BS3 crosslinkers (ProteoChem) that were freshly dissolved in sodium phosphate and added to a final concentration of 1 mM. The crosslinking reactions and a sodium phosphate negative control were incubated at room temperature for 1 hour. To quench the reactions, Tris buffer was added to a final concentration of 60 mM and the samples were maintained at room temperature for 15 minutes. The excess crosslinker was removed via size exclusion chromatography. Samples were then precipitated with 15% trichloroacetic acid, washed with acetone, and re-suspended in a urea solution. Samples were then reduced with dithiothreitol and alkylated with iodoacetamide.

### 2.7. Mass spectrometry and analysis

Samples were digested in solution with trypsin/Lys-C. After digestion, the solution was dried and reconstituted to 1μg/μl in 5% acetonitrile/water (0.1% formic acid). The peptides were then separated by liquid chromatography (Thermo Scientific EASY nLC-1200 coupled to a Thermo Scientific Nanospray FlexIon source) using a pulled glass emitter 75um X 20 cm (Agilent capillary, part#160-2644-5), with the tip packed with Agilent SB-C18 Zorbax 5um packing material (part #820966–922) and the remaining emitter packed with nanoLCMS Solutions UChrom C18 3um packing material (part #80002) and analyzed by MS/MS on a Thermo Scientific Q Exactive Hybrid Quadrupole-Orbitrap Mass Spectrometer.

The raw data were analyzed using Thermo Scientific’s Proteome Discoverer Software. The data was searched using Mascot and Sequest HT against the Rad5 sequence [43,44]. Identification of crosslinks was carried out using Byonic (Protein Metrics) [45]. The following parameters were used for searching the Thermo raw files in Byonic: Cleavage residues were set to RK and digest cutter was set to C-terminal cutter. Peptide termini were set to Fully Specific and maximum number of missed cleavages was set to 3. Fragmentation type was set to QTOF/HCD with a precursor tolerance of 100 ppm, and a fragment tolerance of 0.02 Da. Precursor isotope off by x was set to off by one or two. Protein FDR was set to 1% FDR. Crosslinks were enabled as appropriate for each sample. The crosslinking candidates were filtered by Xlink score. Candidates with positive Xlink scores, Byonic scores above 200, and PEP2D scores below .001 were selected as likely crosslinking sites. Candidates containing consecutive peptides were not considered in analysis.

### 2.8. Accession numbers

The SAXS data, all of the individual snapshots from the triplicate LD simulations, and a description of all relevant methods have been deposited in the SASBDB under the accession code SASDG25.

## 3. Results

### 3.1. Initial Langevin dynamics simulations of Rad5

We carried out LD simulations of Rad5 to better understand its conformational flexibility. Because there are currently no high-resolution structures of Rad5, we used homology modeling to build the starting model for the simulations (Fig 2). We used SWISS-MODEL [46] to generate a homology model of the Rad5 HIRAN domain that was based on the X-ray crystal structure of the HIRAN domain of HLTF (4XZG.pdb) [18] as well as a homology model of the Rad5 RING domain that was based on the structure of the RING domain of PRC1 (4R8P.pdb) [31]. We used Phyre2 [38] to generate a homology model of the Rad5 helicase domain that was based on the structures of the helicase domains of Rad54 (1Z3I.pdb) [32], SWR1 (6GEJ.pdb) [33], Chd1 (3MWY.pdb and 5O9G.pdb) [34,35], INO80 (6FML.pdb) [36], and Mot1 (6G7E.pdb) [37]. Phyre2 was also used to identify nine putative α-helices within the intrinsically disordered regions of Rad5. These putative helices were included in the starting model because our previous studies of non-classical polymerase pol η showed that the inclusion of such putative α-helices substantially improves the agreement between simulations and experimental X-ray scattering data [24]. The starting model also contained an N-terminal 6xHis tag and a C-terminal Twin-Strep tag.

Simulating Rad5 at full atomic resolution is not currently feasible. Thus, the starting model was coarse-grained such that, depending on the size, shape, and charge of the residue in question, each amino acid residue was replaced by one to four pseudo-atoms [41]. The LD simulations were carried out in duplicate using *uiowa_BD* as previously described [24]. Briefly, the positions of each pseudo-atom were calculated using 125 fs time steps and snapshots (PDB files) were recorded every ns for 5 μs of simulation time. These simulations each generated ensembles containing 5,000 individual structures. To ensure sufficient sampling of conformational space, the R_g_ and D_max_ values for the individual structures in the ensembles were graphed as a function of time (Fig 2). We used CRYSOL to generate scattering curves for all of the individual structures [42], and these were averaged to obtain theoretical scattering curves for the full ensembles (see below). The R_g_ and D_max_ values for the full ensembles for this starting model are 62.9 Å and 229 Å, respectively (Table 1).

**Table 1:** SAXS and simulation parameters.

	R_g_ (Å)	D_max_ (Å)	χ^2^
Experimental SAXS data	47.0	178	N/A
Initial simulations	62.9	229	3.73
Refined simulations (ClusPro)	48.5	182	1.74
Refined simulations (ZDOCK)	49.6	192	1.21

To visualize the structures that constitute the full ensemble, we ordered the 5,000 individual structures in order of increasing R_g_. The individual structures corresponding to the 20^th^, 40^th^, 60^th^, and 80^th^ percentiles are shown in Fig 2. Overall, these simulations show that Rad5 possesses a high degree of conformational flexibility. Its flexible N-terminal tail region, which includes the HIRAN domain, samples a wide range of conformations. Interestingly, the HIRAN and helicase domains are in contact or in close proximity with each other (within 6 Å) in approximately 15% of the individual structures in the ensemble. These periods of contact or close proximity persist from as short as 2 ns to as long as 200 ns.

### 3.2. Small-angle X-ray scattering of Rad5

**Fig 3:**
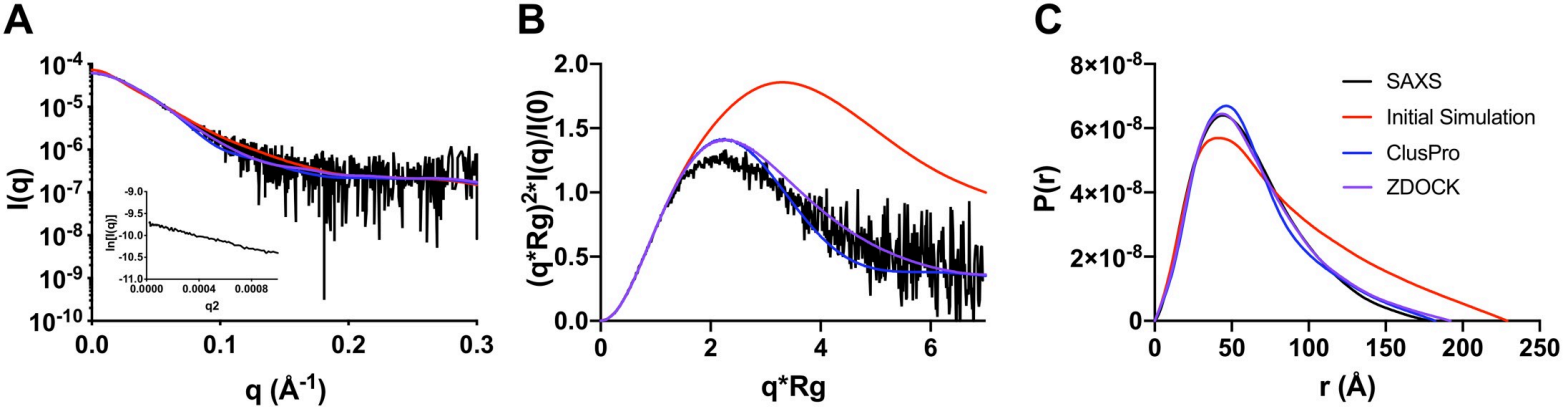
SEC-SAXS analysis of Rad5. (**A**) Experimental SAXS scattering curve (*black*) overlaid with theoretical scattering curves generated from full ensemble simulations of the initial simulations of Rad5 (*red*), as well as two refined simulations utilizing ClusPro (*blue*) or ZDOCK (*purple*). (**B**) Dimensionless Kratky plots from the experimental data (*black*), the initial simulation (*red*) and the two refined simulations (*blue* and *purple*). (**C**) Pairwise distribution plots from the experimental data (*black*), the initial simulation (*red*) and the refined simulations (*blue* and *purple*).

Rad5 containing an N-terminal 6xHis tag and a C-terminal Twin-Strep tag was overexpressed in *E*. *coli* and purified using a Strep-Tactin XT affinity column and Superdex 200 size exclusion column. To experimentally validate the LD simulations, we carried out size exclusion chromatography (SEC)-SAXS (S1 Table and S1 Fig). Buffer subtraction was performed using BioXTAS RAW [27] to obtain an experimental scattering curve (Fig 3). We compared this experimental scattering curve to the theoretical scattering curve obtained from the initial LD simulations using the equation described previously [42], and found that the agreement was rather poor with a χ^2^ equal to 3.73. We used PRIMUS and AUTORG to carry out Guinier analysis and to obtain an experimental R_g_ [28,29]. The linear Guinier plot (Fig 3) shows that the protein is free from aggregation, and the experimental R_g_ is equal to 47.0 Å (Table 1). This experimental value is considerably less than the theoretical R_g_ obtained from the initial simulations (62.9 Å).

The experimental, dimensionless Kratky plot (Fig 3) suggests that Rad5 behaves as a partially disordered protein in solution. The theoretical, dimensionless Kratky plot derived from the initial simulations suggests a greater degree of disorder and conformational flexibility than is observed with the experimental data. Furthermore, the experimental pairwise distribution plot (Fig 3) generated using GNOM [29,30] yielded a D_max_ equal to 178 Å (Table 1). This experimental value is considerably less than the theoretical D_max_ obtained from the initial simulations (229 Å). Moreover, the theoretical, pairwise distribution plot shows a greater degree of extension than is observed with the experimental data. Overall, these results imply that the initial simulations over-estimated the degree of disorder and conformational flexibility in Rad5.

### 3.3. Refined Langevin dynamics simulations of Rad5

To bring the LD simulations of Rad5 into better agreement with the results from the SAXS experiments, we repeated the LD simulations with adjustments made to the starting model. We built two new starting models, both of which had an interaction between the HIRAN domain and the helicase domain. This hypothetical intra-molecular interaction was chosen for three reasons. First, the SAXS results showed that Rad5 is less extended and more compact than the initial LD simulations suggested. Second, we extracted the 58 best-fit structures from the initial ensemble (those with χ^2^ values less than 1.22), and all of them featured the HIRAN and helicase domains in contact or in close proximity. Third, 15% of the individual structures in the initial ensemble had these domains in contact or close proximity.

**Fig 4:**
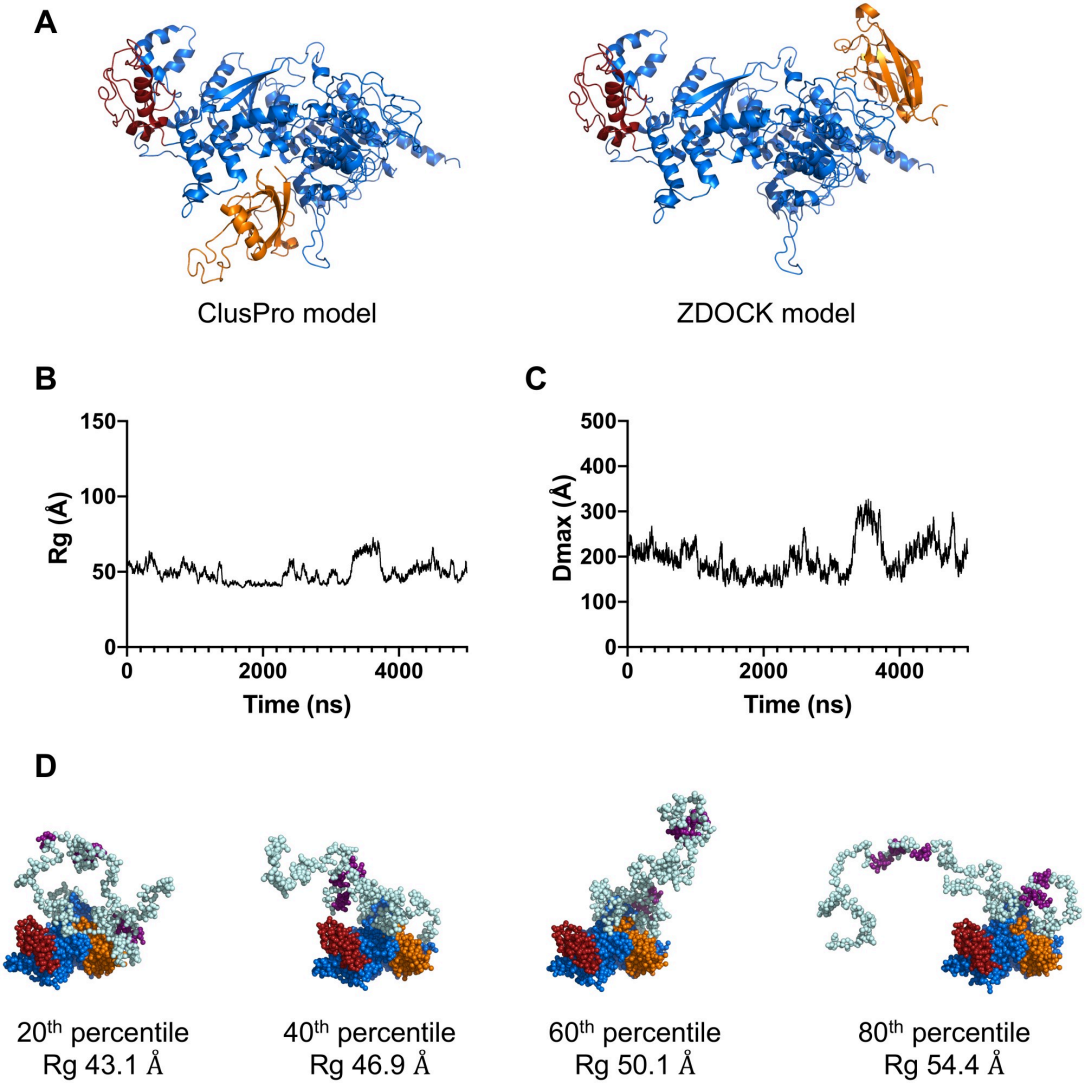
Refined LD simulations of Rad5. (**A**) Refined starting models built using the ClusPro server (*left*) or the ZDOCK server (*right*). The helicase domain is shown in *blue*, the HIRAN domain is shown in *orange*, and the RING finger domain is shown in *red*. The disordered regions have been removed for clarity. (**B**) R_g_ plotted over time for one 5 μs refined ClusPro simulation of Rad5. (**C**) D_max_ plotted over time for one 5 μs refined ClusPro simulation of Rad5. (**D**) Individual structures of Rad5 from the refined ClusPro simulation representing the 20^th^, 40^th^, 60^th^, and 80^th^ percentile of R_g_ values.

To build these new starting models, we used two docking servers (ClusPro and ZDOCK) to generate two different docked poses of the HIRAN domain on the helicase domain (Fig 4) [39,40]. Distance restraints were not imposed with ClusPro in order to ensure that the placement of the HIRAN domain on the helicase domain was not biased by the initial LD simulations. By contrast, distance restraints were imposed with ZDOCK to ensure that the HIRAN domain was placed on the helicase domain in the most common position observed in the initial LD simulations. We used both approaches in order to determine whether the position of the HIRAN domain on the helicase domain would significantly affect the R_g_ and D_max_ values as well as the quality of the fit.

As with the initial LD simulations, these two new starting models were coarse-grained such that each amino acid residue was replaced by one to four pseudo-atoms. The two sets of refined LD simulations were carried out in triplicate using 125 fs time steps. Snapshots were recorded every ns for 5 μs of simulation time, such that each simulation generated an ensemble containing 5,000 individual structures. The R_g_ and D_max_ values for the individual structures in the ClusPro-derived ensemble were graphed as a function of time (Fig 4). The corresponding graphs for the ZDOCK-derived ensemble are shown in S2 Fig. We generated scattering curves for all of the individual structures in a given ensemble, and these were averaged to obtain theoretical scattering curves for the full ensemble. The R_g_ and D_max_ values for the ClusPro-derived full ensemble are 48.5 Å and 182 Å, respectively (Table 1). The R_g_ and D_max_ values for the ZDOCK-derived full ensemble are 49.6 Å and 192 Å, respectively (Table 1). The values from these refined Rad5 LD simulations are in much better agreement with the experimentally derived values than are the values from the initial LD simulations.

We compared the theoretical scattering curves for both the ClusPro-derived ensemble and the ZDOCK-derived ensemble with the experimental scattering curve (Fig 3). We found that the agreement using both starting models was significantly improved compared to the initial simulation. The ClusPro-derived ensemble had a χ^2^ equal to 1.74 and the ZDOCK-derived ensemble had a χ^2^ equal to 1.21. The lower χ^2^ value with the ZDOCK-derived ensemble reflects a better fit of the theoretical and experimental scattering curves in the intermediate q region. Overall, the R_g_ and D_max_ values for the ClusPro-derived ensemble agree with the experimental values marginally better than the ZDOCK-derived ensemble values do, while the scattering curve for the ZDOCK-derived ensemble agrees with the experimental curve marginally better than the ClusPro-derived ensemble does. Given these results and the resolution limitations of SAXS, it is extremely difficult to predict the precise position and orientation of the HIRAN domain on the helicase domain. Nevertheless, these results provide strong support for an intra-molecular interaction between the HIRAN and helicase domains of Rad5.

**Fig 5:**
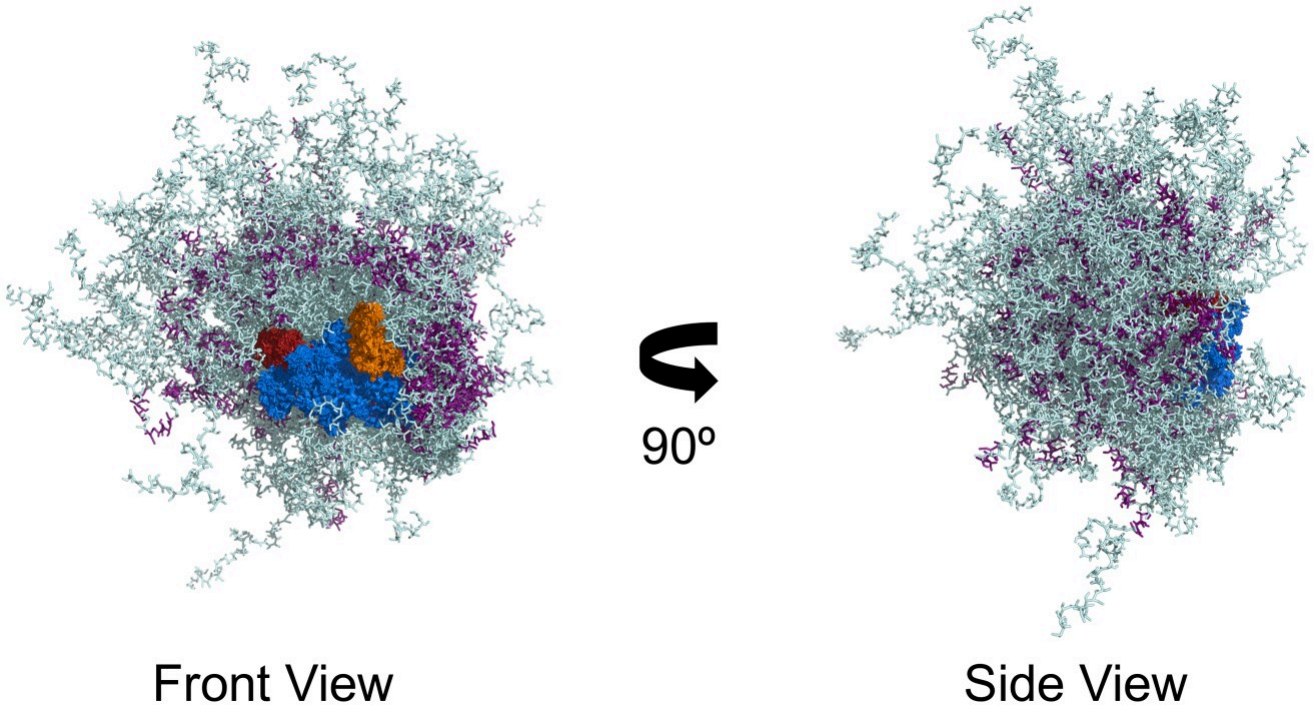
Conformational flexibility of Rad5. Overlay of 100 random individual structures from the refined ClusPro simulation. The helicase domain is shown in *blue*, the RING finger domain is shown in *red*, the HIRAN domain is shown in *orange*, the nine, putative α-helices are shown in *purple*, and the disordered regions are shown in *light blue*. Rad5 can sample a wide range of conformational space, mostly due to the intrinsically disordered N-terminal tail and the intrinsically disordered region between the HIRAN and helicase domains.

We also generated theoretical dimensionless Kratky plots and pairwise distribution plots for both the ClusPro-derived ensemble and the ZDOCK-derived ensemble and compared these with the corresponding experimental plots (Fig 3). The plots from both of these refined Rad5 LD simulations agree better with the experimental plots than do the plots from the initial Rad5 LD simulations. Finally, to visualize the structures that constitute the full, refined ensembles, we ordered the 5,000 individual structures in order of increasing R_g_. The individual structures from the ClusPro-derived ensemble corresponding to the 20^th^, 40^th^, 60^th^, and 80^th^ percentiles are shown in Fig 4. The corresponding structures for the ZDOCK-derived ensemble are shown in S2 Fig. Overall, these simulations show that, while the HIRAN domain is bound to the helicase domain, Rad5 still possesses a high degree of conformational flexibility. This flexibility is mainly in the N-terminal tail region (approximately residues 1 to 170) and the region between the HIRAN domain and the helicase domain (approximately residues 300 to 430) (Fig 5).

### 3.4. Crosslinking the Rad5 HIRAN and helicase domains

**Fig 6:**
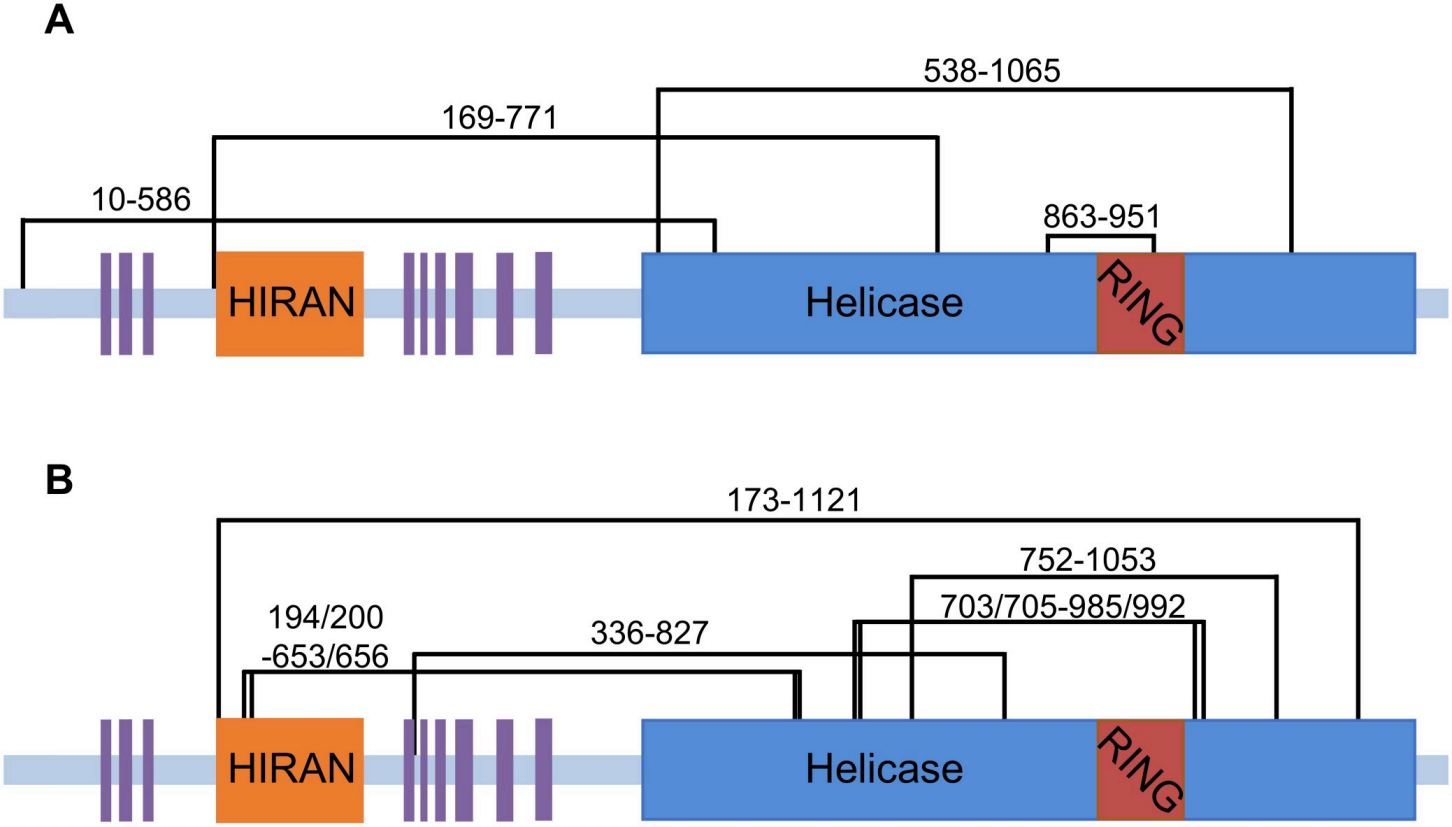
Crosslinking the Rad5 HIRAN and helicase domains. (**A**) Black lines represent the most statistically significant crosslinks in full length Rad5 using the BS^2^G crosslinker. Shown are the helicase domain (*blue*), the RING finger domain (*red*), the HIRAN domain (*orange*) and the nine, putative α-helices (*purple*). Disordered regions are shown in *light blue*. (**B**) Black lines represent the most statistically significant crosslinks in full length Rad5 using the BS3 crosslinker. The structured and disordered regions of Rad5 are depicted as described above.

To provide independent, experimental evidence that the Rad5 HIRAN and helicase domains interact, we carried out crosslinking followed by peptide analysis by mass spectrometry (S2 Table and S3 Fig). The Rad5 protein was treated with homobifunctional crosslinking agents BS^2^G or BS3. The crosslinked protein was precipitated, washed, re-suspended, and digested with trypsin and Lys-C. The desalted peptides were subjected to LC-MS/MS. From the BS^2^G experiment, one crosslink was identified between Lys-169 of the HIRAN domain and Lys-771 of the helicase domain (Fig 6). From the BS3 experiment, two peptide pairs containing a total of three crosslinks were identified. Lys-173 of the HIRAN domain was crosslinked to Lys-1121 of the helicase domain, and Lys-194 and Lys-200 of the HIRAN domain were crosslinked to Lys-653 and Lys-656 of the helicase domain (Fig 6). Most of the remaining crosslinks from both experiments were between residues within the helicase domain.

From our crosslinking experiments, three peptide pairs containing four individual crosslinks between the HIRAN and helicase domains were identified. While it is technically possible that these represent inter-molecular crosslinks between two Rad5 molecules, this is unlikely because multi-angle light scattering (MALS) measurements show Rad5 to be a monomer in solution (S4 Fig). The locations of two of these crosslinking sites on the helicase domain, Lys-771 from the BS^2^G experiment and Lys-1121 from the BS3 experiment, are in close proximity to the docking position generated by ZDOCK that was used in our refined LD simulations. The locations of the other two crosslinking sites, Lys-653 and Lys-656, were identified in a single peptide pair from the BS3 experiment and are in proximity to the position generated by ClusPro that was used in our refined simulations.

## 4. Discussion

The bypass of DNA damage during DNA replication is critical to reducing the frequency of mutagenesis and avoiding genome instability. Rad5 plays two important roles in DNA damage bypass. It functions as an E3 ubiquitin ligase to catalyze the poly-ubiquitylation of PCNA, and it functions as a fork-remodeling helicase to catalyze the conversion of stalled replication forks to chicken foot structures. To begin to understand the structure and conformational flexibility of Rad5, we have employed a full ensemble hybrid method that combines molecular simulations with SAXS [26].

Full ensemble hybrid methods have substantial advantages over the more widely used minimal ensemble hybrid methods [26]. Traditionally, molecular simulations are used to generate a large ensemble of structures. These structures are used in a minimal ensemble search to obtain the fewest number of structures that best fit the experimental SAXS data. These minimal ensembles are highly unrealistic in that they represent the conformational flexibility of a protein by typically only two to four individual structures. By contrast, full ensemble methods are far more realistic in that they represent the conformational flexibility of a protein by thousands of individual structures, each related to one other by a series of time steps in a molecular simulation. In full ensemble methods, the simulations are generally not used to fit the experimental SAXS data [26]. Instead the experimental SAXS data is used simply to validate the simulations.

The present study of the conformational flexibility of Rad5 extends beyond prior full ensemble hybrid studies. This is because there are no experimentally determined structures of any region of Rad5 upon which to build starting models for the LD simulations. For this reason, we had to rely on disorder predictions and on homology modeling. Despite these obvious limitations, we still achieved the same remarkable agreement between the experimental SAXS data and the LD simulations as in prior studies with other proteins [24,25,47]. This remarkable agreement is a testament to the strength of the full ensemble hybrid approach, the accuracy of the LD simulations, the reliability of protein disorder prediction methods, and the accuracy of homology modeling methods.

Our initial LD simulations of Rad5 placed few constraints on the structure of Rad5. It enforced the structure of the three folded domains as well as the putative α-helices within the disordered regions. Interestingly, by comparing the theoretical R_g_ and D_max_ values derived from these simulations with the experimental R_g_ and D_max_ values obtained from the SAXS data, we found that Rad5 is actually more compact and less extended than indicated by the initial simulations. This suggested that Rad5 has an intra-molecular interaction that limits the range of conformational space it can sample to less extended states.

Given these considerations, we carried out refined LD simulations of Rad5 by placing one additional constraint on the starting model: an interaction between the HIRAN and helicase domains. While other intra-molecular interactions would also limit the range of conformational space to less extended states, we chose this particular one because it was the simplest given our knowledge of Rad5 structure. We positioned the HIRAN domain in two different locations on the helicase domain (one derived from ClusPro and the other from ZDOCK) to see if we could discriminate between these two models. We achieved excellent agreement between both of these refined LD simulations of Rad5 and the experimental SAXS data. We were not, however, able to discriminate between the two models. This is probably due to the resolution limitations of the experimental SAXS data. Thus, while one can conclude that the HIRAN and helicase domains likely interact with each other, we must await a high-resolution structure of Rad5 to understand the precise nature of this interaction.

**Fig 7:**
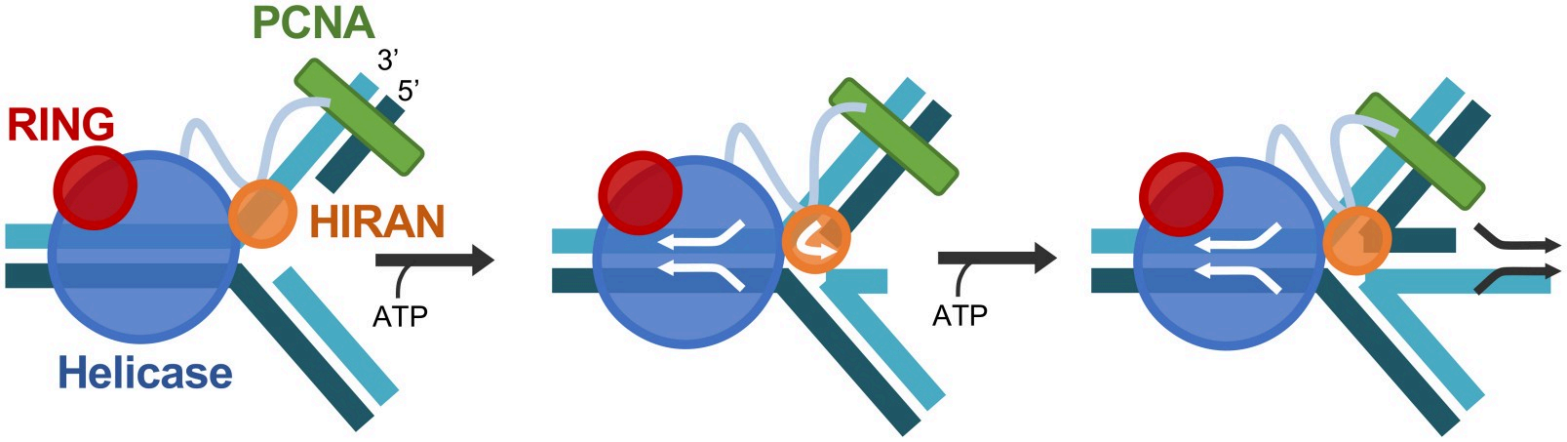
Model of Rad5-catalyzed fork re-modeling. Illustration of a model of Rad5 converting a stalled replication fork to a chicken foot intermediate. Here, a replication fork that has stalled because of damage on the leading strand is shown with Rad5 bound to PCNA (*green*) via the Rad5 N-terminal tail. The RING domain is *red*, the helicase domain is *blue*, and the HIRAN domain is *orange*. ATP hydrolysis facilitates translocation of double-stranded DNA, which leads to unwinding of the replication fork. Arrows indicate the direction in which Rad5 moves the DNA strands. The HIRAN domain binds the 3’ end of the newly synthesized leading strand and redirects its pair with the newly synthesized lagging strand to form the chicken foot structure.

Further support for an intra-molecular interaction between the HIRAN and helicase domains comes from the crosslinking/mass spectrometry data, which shows direct interactions between the HIRAN and helicase domains. This has several important biological and mechanistic implications. First, the Rad5 helicase domain is a Swi/Snf superfamily 2 helicase. These helicase domains are believed to bind to and translocate along double-stranded DNA [48]. One key feature of superfamily 2 helicases is the presence of accessory domains that are associated with the helicase domain that gives each helicase in this superfamily its distinct function [48]. The association between the HIRAN and helicase domains of Rad5 suggests that the HIRAN domain may be the critical accessory domain needed for fork re-modeling. Support for this comes from the fact that the HIRAN domain binds the 3′ end of the DNA, presumably at the end of the primer strand on the leading strand of the replication fork [18]. It is possible that the HIRAN domain binds the primer terminus, while the helicase core domain translocates along the double-stranded DNA directly in front of the replication fork. This would result in the unwinding and peeling back of the primer stands from both the leading and lagging strands and would result in the formation of the chicken foot intermediate and regression of the replication fork (Fig 7). It should be pointed out, however, that this model is speculative as it is based full ensemble studies of Rad5 performed in the absence of the DNA substrate.

Second, Rad5 contains a PIP (PCNA-interacting protein)-like motif, which binds non-classical polymerase Rev1, a common protein present at stalled replication forks, and which likely binds PCNA [49]. This PIP-like motif (residues 9 to 17) is located at the beginning of the N-terminal disordered region of Rad5 [50]. As we have shown, this region has high conformational flexibility (Fig 5). Thus, Rad5 would be able to maintain protein-protein interactions with Rev1 or with PCNA without having significant constraints placed on the position and orientation of its catalytic core. For example, this N-terminal region could act as a tether providing the necessary flexibility to allow Rad5 first to participate in the poly-ubiquitylation of PCNA and then to move to the fork junction to participate in fork re-modeling without having to dissociate from Rev1, PCNA, or any other binding partner. In this way, the conformational flexibility of Rad5 may be critical to regulating and coordinating the two activities of this protein in template switching.

## Supporting information

S1 Table SAXS data collection and analyses. (PDF)

S2 Table Crosslinking results and analyses. (PDF)

S1 Fig The size exclusion chromatography elution profile for SEX-SAXS. The integrated intensity is shown in blue. The Rg values for frames used in further analysis shown in black.
(PDF)

S2 Fig ZDOCK-based simulation of Rad5. (**A**) Rg is plotted as a function of time for a 5 μs simulation. (**B**) Dmax is plotted as a function of time for a 5 μs simulation. (**C**) Individual structures from the resulting ensemble arranged in order of increasing Rg values.
(PDF)

S3 Fig Crosslinks between the helicase domain and the HIRAN damonain of Rad5. The crosslinks in proximity to the ZDOCK position, which is shown in light pink, are indicated in dark pink. Crosslinks in proximity to the ClusPro position, which is shown in light green, are indicated in dark green.
(PDF)

S4 Fig Multi-angle light scattering (MALS) analysis of Rad5. The A280 is shown in black. The molar mass for peak 1 shown in red. The calculated molecular weight is 140 kDa, which is in close agreement with the expected molecular weight of the Rad5 monomer, which is 134 kDa.
(PDF)

## Abbreviations

D_max_: maximal distance
HIRAN: HIP116 Rad5p N-terminal
HLTF: helicase-like transcription factor
LD: Langevin dynamics
MALS: multi-angle light scattering
MD: molecular dynamics
PCNA: proliferating cell nuclear antigen
PIP: PCNA-interacting protein
pol η: DNA polymerase eta
R_g_: radius of gyration
RING: really interesting new gene
SAXS: small-angle X-ray scattering
SEC: size exclusion chromatography
SHPRH: SNF2 histone linker PHD RING helicase
SUMO: small ubiquitin-like modifier
