# Supplementary material for: Conformational flexibility of fork-remodeling helicase Rad5 shown by full-ensemble hybrid methods
Source: PLoS One. 2019 Oct 18;14(10):e0223875. doi: 10.1371/journal.pone.0223875 (PMC6799953; doi:10.1371/journal.pone.0223875)
Supplement: S1 Table — (PDF) [file pone.0223875.s001.pdf]

Table S1. SAXS data collection and analyses

**(a) Sample details**	Rad5
Organism	Saccharomyces *cerevisiae*
Source	Escherichia *coli* expressed
Construct Description	Full length Rad5 (His- and Twin-Strep-tagged)
Extinction coefficient	124,720 M^{-1} cm^{-1}
M from chemical composition	137,694 Da
Sample volume, concentration, flow rate	175 µl, 7.75 mg/ml, 0.7 ml/min
FPLC and Column	AKTA Pure, Superdex 200 Increase 10/300 GL column
Solvent details	40 mM Tris pH 8.0, 150 mM KCl, 5 mM DTT, 5% glycerol

**(b) SAXS data collection parameters**	Rad5
X-ray beam Source	BioCAT facility at the Advanced Photon Source beamline 18ID
Detector (Distance)	Pilatus3 1M (Dectris) detector (3.5 m)
Wavelength	1.033 Å (12KeV)
Beam size	150 (h) x 25 (v) μm^2
q-measurement range	0.004-0.4 $Å^{-1}$
Absolute scaling method	N/A
Basis for normalization to constant counts	To incident intensity, by ion chamber counter
Method for monitoring radiation damage	Automated frame-by-frame comparison of relevant regions
Exposure time, number of exposures	0.5 s exposure with a 2 s total exposure period (0.5 s on, 1.5 s off) of entire SEC elution
Sample configuration	SEC-SAXS. Size separation by an AKTA Pure with a Superdex 200 Increase 10/300 GL column. SAXS data measured in a 1.5 mm ID quartz capillary.
Sample temperature	20°C

**(c) Software employed for SAXS data reduction**	Rad5
SAXS data reduction and solvent subtraction	Radial averaging; frame comparison, averaging, and subtraction done using BioXTAS RAW 1.6.0 (Hopkins *et al.,* 2017)
Basic analyses: Guinier Rg and P(r)	Guinier fit using BioXTAS RAW 1.6.0, P(r) function using GNOM (Svergun, 1992)

**(d) Structural parameters**	Rad5
Guinier Analysis	
I(0)	0.00006043 +/- 0.0000002302
R_g	47.0079 +/- 0.3058
qR_g range	0.2704 - 1.2842
Coefficient of correlation, R^2	0.9877
P(r) analysis	
I(0)	0.000061 +/- 0.000000232
R_g	49.18 +/- 0.3102
D_{max}	178
q range	0.0058 - 0.3627
X^2 (total estimate from GNOM)	1.0914
