# Supplementary material for: Conformational flexibility of fork-remodeling helicase Rad5 shown by full-ensemble hybrid methods
Source: PLoS One. 2019 Oct 18;14(10):e0223875. doi: 10.1371/journal.pone.0223875 (PMC6799953; doi:10.1371/journal.pone.0223875)
Supplement: S2 Table — (PDF) [file pone.0223875.s002.pdf]

S4. Supplemental Table 2: Crosslinking results and analyses.

Cross-linking agent	Peptide 1 sequence	Peptide 2 sequence	Cross-link position peptide 1	Cross-link position peptide 2	Score	Xlink Score	PEP 2D	PEP 1D	FDR 1D	q-value 1D
BS2G	**K**RFFNDDLDTSETSLNFK	PYAS**K**	10	586	360.5	45.4	1.90E-05	6.70E-05	0	1.10E-06
BS2G	LED**K**VTWKRFIGALQVTGMATRPTVR	RT**K**	169	771	587.1	25.4	1.20E-10	4.40E-10	0	1.90E-11
BS2G	GGILSDEMGLG**K**TVAAYSLVLSCPHDSDVVDKK	TSVLADFAV**K**	538	1065	416.8	23.1	0.00014	0.0005	0	6.40E-06
BS2G	**K**RFFNDDLDTSETSLNFK	PYAS**K**	10	586	351.8	14.6	9.10E-05	0.00032	0	4.60E-06
BS2G	LED**K**VTWKRFIGALQVTGMATRPTVRPLK	RT**K**	169	771	444.1	11.6	1.70E-08	5.90E-08	0	1.50E-09
BS2G	LED**K**VTWKRFIGALQVTGMATRPTVRPLK	RT**K**	169	771	415.1	11.3	1.80E-08	6.20E-08	0	1.60E-09
BS2G	NN**K**LVTEQTVELDSLMR	CLFEYIEFQNS**K**NLGLK	863	951	394.1	8.9	3.10E-07	1.10E-06	0	2.50E-08
BS2G	**K**RFFNDDLDTSETSLNFK	PYAS**K**	10	586	363.2	1.5	1.90E-06	6.70E-06	0	1.40E-07
BS3	PTVRPL**K**YGSQM**K**	TPPTVVLTTYGIVQNEWT**K**HS**K**	194, 200	653, 656	274.8	70.2	0.00045	0.00086	0.00013	0.00013
BS3	S**K**R	**K**YSTILVHILRLRQVCCHPGLIGSQDENDEDLSK	336	827	379.1	68.1	0.00021	0.0004	0	6.10E-06
BS3	S**K**R	**K**YSTILVHILRLRQVCCHPGLIGSQDENDEDLSK	336	827	405.8	61.9	9.10E-05	0.00018	0	3.00E-06
BS3	AVMALQG**K**C**K**	NLEF**K**PYSPAS**K**	703, 705	985, 992	460.6	54.7	1.60E-06	3.10E-06	0	6.50E-08
BS3	NY**K**QAFDVVNAILEPVLLR	FDGRLSL**K**ER	752	1053	250.9	29.7	4.30E-06	8.30E-06	0	1.60E-07
BS3	TKTPPTVVLTTYGIVQNEWT**K**HS**K**	PTVRPL**K**YGSQM**K**LK	653, 656	194, 200	210.8	19.4	0.00026	0.00049	0	7.80E-06
BS3	IGQTNSV**K**	VTW**K**RFIGALQVTGMATRPTVRPLK	1121	173	383	3.5	1.60E-05	3.10E-05	0	6.30E-07
BS3	IGQTNSV**K**	VTW**K**RFIGALQVTGMATRPTVRPLK	1121	173	325.6	2.3	5.90E-06	1.10E-05	0	2.10E-07
BS3	IGQTNSV**K**	VTW**K**RFIGALQVTGMATRPTVRPLK	1121	173	314.4	0.9	0.00027	0.00051	0	8.30E-06
