# Supplementary material for: Conformational flexibility of fork-remodeling helicase Rad5 shown by full-ensemble hybrid methods
Source: PLoS One. 2019 Oct 18;14(10):e0223875. doi: 10.1371/journal.pone.0223875 (PMC6799953; doi:10.1371/journal.pone.0223875)
Supplement: S1 Fig — The integrated intensity is shown in blue. The Rg values for frames used in further analysis shown in black. (PDF) [file pone.0223875.s003.pdf]

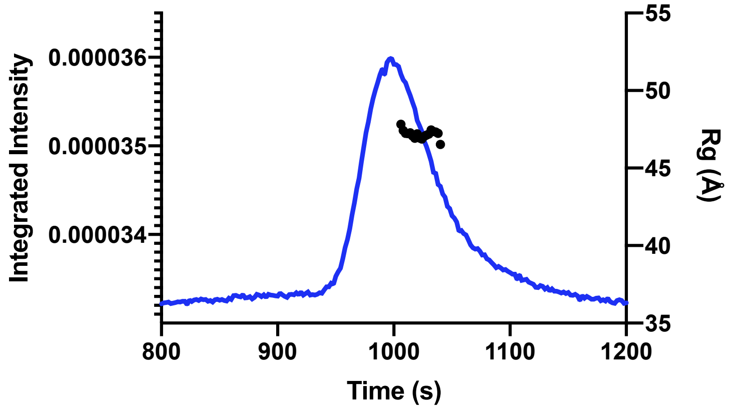

S2. Supplemental Figure 1: The size exclusion chromatography elution profile for SEC-SAXS. The Integrated intensity is shown in *blue*. The R_g values for frames used in further analysis shown in black.
