# Supplementary material for: Conformational flexibility of fork-remodeling helicase Rad5 shown by full-ensemble hybrid methods
Source: PLoS One. 2019 Oct 18;14(10):e0223875. doi: 10.1371/journal.pone.0223875 (PMC6799953; doi:10.1371/journal.pone.0223875)
Supplement: S2 Fig — (A) Rg is plotted as a function of time for a 5 μs simulation. (B) Dmax is plotted as a function of time for a 5 μs simulation. (C) Individual structures from the resulting ensemble arranged in order of increasing Rg values. (PDF) [file pone.0223875.s004.pdf]

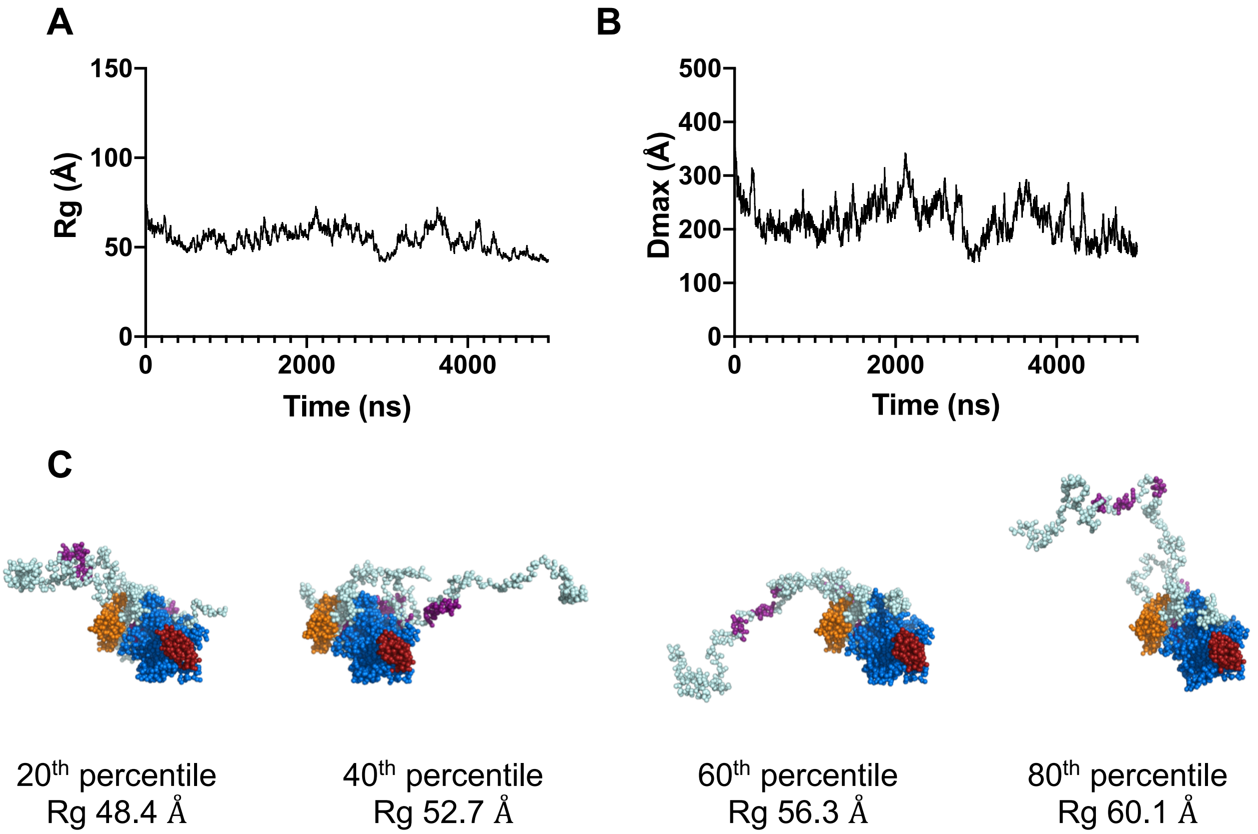

S3. Supplemental Figure 2: ZDOCK-based simulation of Rad5. (**A**) R_g is plotted as a function of time for a 5 µs simulation. (**B**) D_{max} is plotted as a function of time for a 5 µs simulation. (**C**) Individual structures from the resulting ensemble arranged in order of increasing R_g values.
