# Supplementary material for: Conformational flexibility of fork-remodeling helicase Rad5 shown by full-ensemble hybrid methods
Source: PLoS One. 2019 Oct 18;14(10):e0223875. doi: 10.1371/journal.pone.0223875 (PMC6799953; doi:10.1371/journal.pone.0223875)
Supplement: S3 Fig — The crosslinks in proximity to the ZDOCK position, which is shown in light pink, are indicated in dark pink. Crosslinks in proximity to the ClusPro position, which is shown in light green, are indicated in dark green. (PDF) [file pone.0223875.s005.pdf]

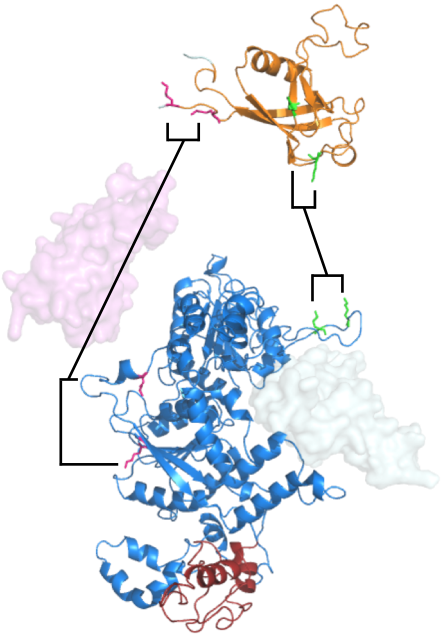

S5. Supplemental Figure 3: Crosslinks between the helicase domain and the HIRAN domain of Rad5. The crosslinks in proximity to the ZDOCK position, which is shown in *light pink*, are indicated in *dark pink*. Crosslinks in proximity to the ClusPro position, which is shown in *light green*, are indicated in *dark green*.
