# Supplementary material for: Conformational flexibility of fork-remodeling helicase Rad5 shown by full-ensemble hybrid methods
Source: PLoS One. 2019 Oct 18;14(10):e0223875. doi: 10.1371/journal.pone.0223875 (PMC6799953; doi:10.1371/journal.pone.0223875)
Supplement: S4 Fig — The A280 is shown in black. The molar mass for peak 1 shown in red. The calculated molecular weight is 140 kDa, which is in close agreement with the expected molecular weight of the Rad5 monomer, which is 134 kDa. (PDF) [file pone.0223875.s006.pdf]

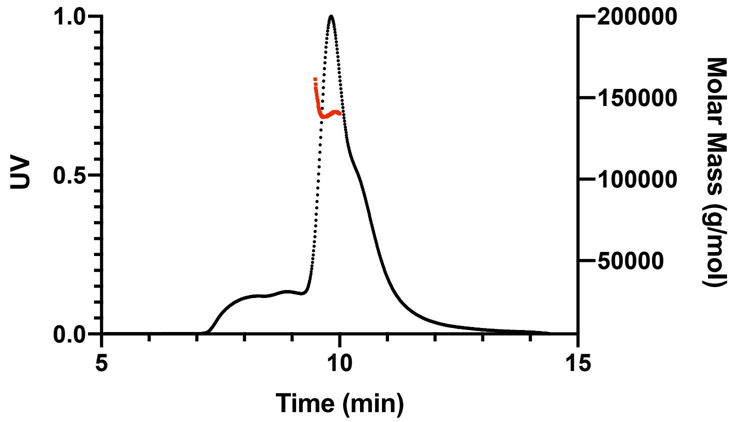

S6. Supplemental Figure 4: Multi-angle light scattering (MALS) analysis of Rad5. The A_{280} is shown in *black*. The molar mass for peak 1 shown in *red*. The calculated molecular weight is 140 kDa, which is in close agreement with the expected molecular weight of the Rad5 monomer, which is 134 kDa.
